# Biphasic Behavior of Anti-M Antibody and Hemolytic Disease of the Fetus and Newborn (HDFN)

**DOI:** 10.7759/cureus.73756

**Published:** 2024-11-15

**Authors:** Mohammad Barouqa, Huseyin Kilic, Nestor Dela Cruz

**Affiliations:** 1 Transfusion Medicine, University of South Alabama College of Medicine, Mobile, USA; 2 Pathology and Laboratory Medicine, University of South Alabama College of Medicine, Mobile, USA

**Keywords:** alloimmunization, anti-m alloantibody, anti-m antibody, blood bank, clinical pathology, fetal doppler ultrasound, hemolytic disease of the fetus & newborn (hdfn), immunohematology, maternal alloimmunization, transfusion medicine

## Abstract

The anti-M antibody is a cold, naturally occurring immunoglobulin M (IgM) antibody that is generally considered clinically insignificant and often overlooked in transfusion practices and assessments of patients at risk for hemolytic disease of the fetus and newborn (HDFN). However, the presence of an IgG component in this case renders the antibody clinically significant, underscoring the necessity for proper serologic testing during prenatal evaluations. We present a case involving an anti-M antibody with an IgG component to highlight the critical importance of thorough serologic testing during prenatal testing.

## Introduction

Hemolytic disease of the fetus and newborn (HDFN) is a significant condition during pregnancy and the neonatal period, primarily resulting from maternal alloimmunization due to exposure to fetal red blood cells that express foreign antigens that are not expressed by maternal red blood cells in addition to exposure to incompatible red blood cells. These events can lead to the formation of immunoglobulin (IgG) antibodies by the maternal immune system, actively transported across the placenta into the fetus, leading to life-threatening hemolysis such as anti-D or suppression of erythropoiesis like anti-K (KEL1) [1,2].

During pregnancy, severely affected fetuses require intrauterine transfusions, and several may require transfusions in the first months of life [3]. The most common antibodies implicated in HDFN are the ABO blood group system and the Rh blood group system, with other blood group systems that include Kidd and Duffy [4].

Anti-M, an antibody in the MNS blood group system, is a naturally occurring antibody, a relatively common and cold alloantibody with an immunoglobulin M (IgM) configuration. It is incapable of passing across the placenta. However, the IgG form of anti-M, although extremely rare, has been reported and found capable of crossing the placenta and causing HDFN [5].

The objective of this case report is to present the significance of prenatal testing for alloantibodies and serologic phenotyping of both parents in patients exhibiting anti-M antibodies with IgG reactivity.

## Case presentation

A 24-year-old patient (G2P1001 or gravidity 2 (two pregnancies including the current one), parity 1001: one prior term delivery, no preterm deliveries, no abortuses, and one living child) at 32 weeks of gestation presented for routine testing and follow-up. She does not have any previous significant medical or surgical history, and she was never transfused any units of blood. Her previous pregnancy was not eventful for any significant complications. While her routine complete blood count showed normal results (Table 1), her type and screen revealed an ABO blood group of "A positive" with a positive antibody screen.

**Table 1:** Results of the routine complete blood count analysis

Parameter	Results	Reference Range
Hemoglobin	11.4 g/dL	9.5–15 g/dL (for the third trimester)
Ferritin	140 ng/mL	30–300 ng/mL
Folic acid	6.1 ng/mL	4.1–10.9 ng/mL
Basic metabolic profile	Normal	-

The screening was initially performed in the solid phase, with one out of three cells showing 2+ reactivity. Accordingly, screening was repeated in a tube with a polyethylene glycol (PEG) enhancer, which showed 2+ reactivity in two out of three screening cells. All the positive screening cells were homozygote (double dose) for the M antigen. Further antibody identification performed in tube testing with PEG revealed a pattern consistent with anti-M antibody reactive at immediate spin and AHG. The antibody was reactive when tested using the prewarm technique, and the patient typed negative for the M antigen serologically. Autocontrol, performed in tube testing with a PEG enhancer, was negative.

Additionally, reactivity was reduced from 2+ to weak reactivity using an enzyme (ficin)-treated reagent. All other significant antibodies were ruled out. Tube testing with LISS (low ionic strength saline) was not performed due to insufficient sample quantity. Testing is summarized in Table 2.

**Table 2:** Summary of serologic testing PEG: polyethylene glycol; AHG: anti-human globulin

Test Name and Methodology	Result	Interpretation of Test
Screening solid phase and tube with PEG	Positive (normal: negative)	Screening is positive and indicates the presence of alloantibody. Further testing is required
Further antibody identification in solid phase and tube with PEG	Positive (normal: negative)	Antibody identified as anti-M, reactive at immediate spin and AHG
Prewarm testing	Positive (normal: negative)	Anti-M reactivity at AHG is confirmed (IgG component is demonstrated)
Serological phenotype	Negative for M antigen	M antigen is not normally expressed on the surface of red blood cells
Enzyme-treated reagent cells	Decreased reactivity (2+ to weak) (expected reactivity for anti-M: decrease in reactivity)	Test supports the presence of anti-M antibody

A 60-minute saline AHG test was performed using reagent cells homozygote (double dose) to the M antigen to record the antibody titers; the titer was recorded at <1. The direct antiglobulin test (DAT) was negative with DAT-monospecific anti-IgG and monospecific anti-C3. Paternal testing to serologically type for the M antigen was not performed. The anti-M antibody titer was monitored weekly and subsequently recorded at 16. Due to this increase in titer (i.e., an increase of more than two test tubes), a Doppler ultrasound of the middle cerebral artery was performed to record the multiples of the median (MoM). The MoM was found to be elevated at 1.60 (normal hemoglobin fetuses: 1.22 ± 0.21) both initially and upon retesting. The patient underwent periumbilical sampling and intrauterine transfusion for fetal anemia.

## Discussion

The M antigen is a human blood group antigen expressed by the glycophorin A (*GYPA*) gene on chromosome 4q31.21 [6]. The majority of anti-M antibodies are cold and clinically insignificant. Hence, they are generally overlooked in transfusion practice, except in specific situations such as hypothermic surgical procedures (e.g., cardiac bypass with cold cardioplegia). When testing for anti-M antibodies, it is extremely important to recognize that the clinically insignificant anti-M antibody can react strongly at room temperature, and leaving the tube to cool after centrifugation and prior to assessing hemagglutination can result in misinterpreting the anti-M antibody as clinically significant or reactive at 37°C.

Additionally, when the anti-M antibody exhibits a high titer or affinity, its reactivity will be strong at room temperature and may persist at 37°C and during the AHG testing phases, potentially leading to the misinterpretation of the anti-M antibody as clinically significant. If an anti-M antibody demonstrates a confirmed reactivity at 37°C either in tube testing or in the solid phase, red cell units that are compatible with M-negative cross-matching are warranted. This confirmed reactivity at 37°C indicates that the anti-M antibody is either demonstrating an IgM form with thermal amplitude or an IgG component that has been reported to possess a complement activating capacity [7].

In addition to anti-N antibodies, anti-M antibodies are characterized by their decreased reactivity with ficin or papain-treated reagent cells, which can be used to support their presence in serum [8]. Although it is rare, HDFN has been previously reported in association with anti-M antibodies (with IgG reactivity) and even related to multiple intrauterine deaths that necessitated maternal treatment, including plasmapheresis treatments, in order to reduce the titer of anti-antibodies [9]. In anti-M-induced HDFN, fetal DAT can be negative, as it is hypothesized that the intravascular hemolysis can be rapid. In contrast, others hypothesized that the clinically significant form of the antibody has the capacity to suppress the erythroid progenitor cells in the bone marrow [10].

Our case shows the importance of evaluating anti-M antibodies, especially when establishing their clinical significance. Furthermore, it is important to demonstrate properly the reactivity of anti-M antibodies at 37°C, regularly monitoring the antibody titers and recording any increase exceeding two tubes, as this might be an indication that the fetus is at risk of HDFN and warrant a Doppler fetal ultrasound of the middle cerebral artery to noninvasively assess fetal anemia.

## Conclusions

This case underscores the critical importance of evaluating and monitoring anti-M antibodies in prenatal care and determining their clinical significance, especially since they have the capacity to demonstrate IgG reactivity in addition to their natural IgM component and can result in HDFN. Patients with anti-M antibodies exhibiting an IgG component should have their antibody titers monitored frequently to identify any increases that may necessitate non-invasive fetal testing, such as middle cerebral artery Doppler ultrasound, to evaluate for fetal anemia. Additionally, patients confirmed to have this antibody component should receive red blood cell units that are M antigen-negative and crossmatch-compatible.
